# The influence of oral vocabulary knowledge on individual differences in a computational model of reading

**DOI:** 10.1038/s41598-023-28559-3

**Published:** 2023-01-30

**Authors:** Ya-Ning Chang

**Affiliations:** 1 Miin Wu School of Computing, National Cheng Kung University, Tainan, Taiwan; 2 MRC Cognition and Brain Sciences Unit, University of Cambridge, Cambridge, UK

**Keywords:** Psychology, Human behaviour

## Abstract

Studies have demonstrated systematic individual differences in the degree of semantic reliance (SR) when reading aloud exception words in adult skilled readers. However, the origins of individual differences in reading remain unclear. Using a connectionist model of reading, this study investigated whether oral vocabulary knowledge may affect the degree of SR as a potential source of individual differences in reading. Variety in oral vocabulary knowledge was simulated by training the model to learn the mappings between spoken and meaning word forms with different vocabulary sizes and quantities of exposure to these vocabularies. The model’s SR in the reading aloud task was computed. The result demonstrated that the model with varying amounts of oral exposure and vocabulary sizes had different levels of SR. Critically, SR was able to predict the performance of the model on reading aloud and nonword reading, which assimilated behavioural reading patterns. Further analysis revealed that SR was largely associated with the interaction between oral vocabulary exposure and oral vocabulary size. When the amount of exposure was limited, SR significantly increased with vocabulary sizes but decreased then with vocabulary sizes. Overall, the simulation results provide the first computational evidence of the direct link between oral vocabulary knowledge and the degree of SR as a source of individual differences in reading.

## Introduction

Learning to read requires readers to master the correspondence between print and sound (orthography-to-phonology, OP), as well as the correspondence between print and meaning (orthography-to-semantics, OS). Over the past few decades, extensive research has been devoted to understanding reading processes^1–10^. Contemporary computational models of reading have reached a consensus that access to sound or meaning can be via multiple pathways, albeit the nature of the detailed procedures remains debatable^4,11^.

Within the connectionist models of reading^4,9,12^, phonology can be accessed either directly via the OP pathway, or indirectly—first activating semantics and then via the semantics-to-phonology (SP) mapping to phonology (i.e., the OSP pathway). On the other hand, semantics can be accessed either directly via the OS pathway, or indirectly—first activating phonology and then via the phonology-to-semantics (PS) mapping to semantics (i.e. the OPS pathway). When readers learn to master the fundamental skills of mapping between representations, the exact pathway used to extract sound and meaning can be flexible. Recent studies have demonstrated systematic individual differences in the degree of semantic reliance (SR) when reading aloud exception words (e.g. *pint, snow*) in adult skilled readers^5,10^. Woollams et al.^10^ showed that individuals with high SR tended to read high imageability words (e.g., *bird, moon*) faster than those with low SR but were slower in nonword reading. It is likely that varying degrees of SR could be associated with individuals’ semantic and phonological processing in the brain. This proposal was corroborated by Hoffman et al.’s^5^ recent neuroimaging data, which demonstrated that during exception word reading, the degree of SR was positively correlated with brain activations in the left anterior temporal lobe (LATL) associated with semantic processing. By contrast, SR was negatively correlated with brain activations in the left precentral gyrus (LPCG) associated with phonological processing. However, the origin of individual differences in the degree of SR during word reading has not been sufficiently investigated.

Another line of studies has investigated individual differences among beginning readers^13–15^. Siegelman et al.^14^ demonstrated that in a large cohort of children, those who were more sensitive to OP than OS regularities in a reading aloud task performed better on a range of reading tasks. Furthermore, sensitivity to OP and OS regularities in children with reading disabilities is a crucial predictor of how much children can benefit from reading intervention^15^. Children relying more on OP regularities and less on OS regularities in pre-intervention phases had better intervention gains. These studies demonstrated that individual differences in reading could be observed in the early years of learning to read. Relatedly, a recent mega study of reading through lifespan demonstrated that individuals’ imageability effects, indexing the involvement of the OS process, decreased with age, and were negatively correlated with their reading aloud skills^13^. The results revealed that poorer readers with low reading aloud skills relied on the OS process more than the OP process compared to good readers.

These studies suggest that individual differences in word reading could be observed for both adults and children. However, what might cause individual differences in reading, especially with respect to the division of labour between OP and OS processes? This is a central issue in the present study. In reading research, several potential sources of individual differences have been considered. For instance, various studies have demonstrated the impact of lexical quality on individual differences in visual word recognition^1,16–19^. With more reading experience, readers can develop more precise and stable word-specific knowledge (i.e., high lexical quality^20^) that supports coherent activation of form, sound, and meaning of a word. Yap et al.^18^ used a measure of vocabulary size as an index of lexical quality in a mega study of reading aloud and lexical decision, demonstrating that vocabulary was associated with variability among individuals’ responses in which higher vocabulary was associated with reduced effects of frequency and OP consistency. Another critical factor from a computational point of view is the relative computational resources in the alternative reading pathways^9,21,22^. If there are imbalanced processing units in the alternative reading pathways, the system would rely on a pathway that has more processing resources, which in turn influences reading behaviours.

While it is relatively clear that reading experience and reading capacity would have an impact on individual differences in reading, not enough is known about the influence of oral language, given that reading is a relatively late human invention and relies heavily on preliteracy language experience. Evidence from both behavioural and computational modelling has demonstrated that oral vocabulary knowledge interacts with reading development^8,23–27^. Particularly, Chang and Monaghan^28^ demonstrated that diversity and quantity of oral language skills could predict reading performance in a triangle model of reading. Given that there is huge variability in children’s language experience prior to learning to read^29–31^, oral vocabulary knowledge could be potentially important to explain individual differences in reading. To date, however, there has not been a systematic investigation of individual differences that are potentially linked to varying degrees of oral vocabulary knowledge except for a recent behavioural study by Siegelman et al.^14^. Siegelman et al.^14^ reported that individual children’s oral vocabulary size had a positive correlation with reliance on OP and their reading outcome and a negative correlation with OS. Moreover, after controlling for oral vocabulary size, significant associations between individual children’s reliance on OP and OS and their reading skills were still observed. The results seem to suggest that the predictabilities of reliance on OP and OS in children’s reading skills could not be entirely attributed to their vocabulary knowledge. However, Siegelman et al.^14^ have focused on investigating children’s oral vocabulary size; the quantity of language exposure to oral vocabulary was not considered.

Thus, the present study was designed to investigate individual differences using computational models of reading, focusing on the influences of both diversity and quantity of oral language skills on the division of labour between OP and OS processes. Computational modelling has played an important role in advancing our understanding of learning to read by making theoretical ideas testable and providing possible mechanistic accounts^4,9,11,12,28^. Models can be designed to reflect the general processing principles in the human reading system ^4^ or to establish a parametric fit to human reading performance^11^. Regardless of approaches to reading, models should be able to reproduce key psycholinguistic effects in word reading, such as frequency^32^, neighbourhood size^33^, and consistency^34^. The present study used the triangle modelling framework because the model includes both oral and written language processing components, allowing for the exploration of mutual influences. Additionally, the model implemented multiple pathways to reading. This flexibility made the model particularly suitable for investigating individual reliance on alternative pathways during reading. Specifically, this study first evaluated whether the present triangle model of reading could reproduce a range of standard reading effects commonly reported in computational modelling studies of reading^4,9,11,23^. Next, the model was used to investigate whether the degree of SR during reading could be directly associated with variations in oral vocabulary size and quantity of oral language exposure, with reading experience and computational capacity being considered. The SR measure was implemented in two ways in the model. One approach, being close to behavioural settings, was derived by investigating the effect size of consistency when reading exception words^10^. Another approach, using the unique advantage of computational modelling, was based on a direct measure of the model’s division of labour on the use of phonological and semantic pathways for reading. Both the SR measures were investigated in terms of their predictabilities on behavioural reading patterns and their relationships with oral vocabulary knowledge in the model.

## Method

The architecture of the model, the procedures of model training, and the performance testing procedures were identical to those used in a previous study^28^. The following sections aimed to provide a brief summary.

### Network architecture

**Figure 1:**
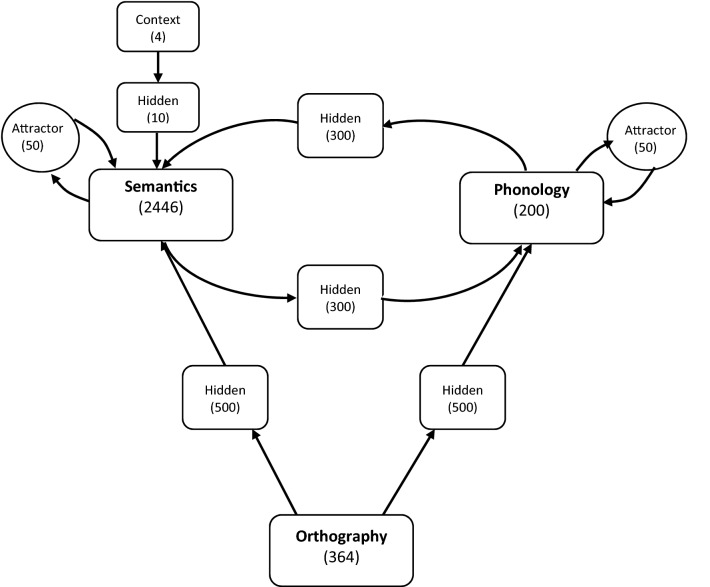
The architecture of the model. Numbers in brackets indicate the number of units in that layer.

The model was a deep and recurrent neural network that leaned the mappings between orthographic forms of words, their sounds, and meanings via intermediate layers, as shown in Fig. 1. Specifically, the model contained three key processing layers (orthography, phonology, and semantics), five intermediate hidden layers, and two attractor layers. The function of the attractor was to help the model develop stable representations of words. Additionally, to differentiate words that had the same orthographic form but more than one meaning (i.e. homophones), the model utilised the information provided by four context units. The context unit assigned to the meaning of each word was randomly selected at the beginning of training. For non-homophones, none of the context units were active. In the model, all units were fully connected from one layer to another.

### Model environment

The model environment contained orthographic, phonological, and semantic patterns for each of 6229 monosyllabic English words. This vocabulary set included the majority of inflected forms of monosyllabic English words. Orthographic patterns of words were represented by 14 letter slots, in which each slot comprised 26 units for each alphabet. Words were aligned with their first vowel on the fifth slot, and the second vowel was placed on the sixth slot if applicable. Consonants preceding or following the vowel(s) were placed in adjacent slots to the two vowel slots. Phonological patterns of words were represented by eight phoneme slots, in which each slot comprised 25 units for phonological features such as voice, nasal, round, etc. The first three phoneme slots were for onset consonants, the fourth slot was for the vowel and the remaining four slots were for coda consonants. Lastly, semantic representations of words were derived from Wordnet^35^, consisting of 2446 semantic features. The representational scheme was binary with one indicating the presence of semantic features and with zero indicating the absence.

### Training

Akin to children’s learning to read, the model was trained with two phases. In oral language training, the model learned the mapping from semantics to phonology (SP), conceived as a meaning naming task, and the mapping from phonology to semantics (PS), conceived as an oral vocabulary comprehension task. In reading training, the model learned the mappings from orthography to both phonology and semantics. To simulate individuals with different levels of the breadth and depth of oral vocabulary, the model was trained to learn the mappings between spoken and meaning word forms with different vocabulary sizes and quantities of exposure to these vocabularies. Importantly, the subsequent reading training procedures were kept the same across conditions, which could isolate the impact of oral vocabulary knowledge on individual differences in reading in the model.

The model’s learning experience in oral language training was manipulated in two ways: (a) the model was trained with six oral vocabulary sizes, 1000 words to 6000 words in a step of 1000 words; (b) the training was conducted with varying amount of exposure, either sampling words 400 K times from the vocabulary, or 800 K, 1.2 M, 1.6 M or 2 M times. The symbols K and M represented 1000 and 1,000,000, respectively. Consequentially, there were 30 oral-language training conditions as a source of variations in oral vocabulary knowledge. The set of words in each vocabulary size was selected from the whole training set based on frequency derived from the Wall Street Journal corpus^36^. For instance, the 1000 vocabulary size condition consisted of the most frequent 1000 words in the language. During oral language training, the input pattern of each word was presented constantly for eight network time steps, and in the last two-time steps, the model was required to reproduce the target pattern. The model additionally learned to develop stable phonological to phonological (PP) and semantic to semantic (SS) representations. The phonological or the semantic representation was presented constantly for two-time steps and then the model cycled activation for further six-time steps to reproduce the initial representation. The four tasks (PS, SP, PP, and SS) were interleaved, with 40% of trials for the oral vocabulary task, 40% of trials for the meaning naming task, 10% of trials for the phonological attractor, and 10% for the semantic attractor. For each trial, a word was randomly selected based on the frequency with replacement. The model was trained with a different number of presentations of words according to its exposure conditions.

After oral language training, the weights of connections were fixed, and then the model was trained to learn to read the whole vocabulary set. The model was presented with orthographic forms of words for 12 network time steps. From the time steps seven to 12, the model was asked to produce target phonological representations as in a reading aloud task, and to produce target semantic representations as in a written word comprehension task. For each reading learning trial, a word was randomly selected according to word frequency. The model was trained up to 1 M reading learning trials and that was applied to each of the 30 oral language conditions of the model.

The model was trained using the back-propagation through time (BPTT) algorithm^37,38^ with a standard learning rate of 0.05. The training parameter was the same for both oral language training and reading training. Four versions of each model with different initial randomised parameters were run to ensure that these random parameters did not adversely affect the simulations. Thus, there were in total 120 simulations of the model (i.e. six vocabulary conditions x five exposure conditions x four versions).

### Testing

The approach to evaluating model performance was adopted from previous simulation work^4,28^. For phonology, error score was measured by summed squared differences between the model’s actual phonological pattern and its target pattern. Accuracy was measured by computing the closest phoneme to the model’s actual output based on the Euclidean distance and determining whether the actual and target phonemes were the same for all phoneme slots. Similarly, for semantics, error score was measured by summed squared differences between the model’s actual semantic pattern and its target pattern. Accuracy was measured by computing the Euclidean distance between the model’s actual semantic representation and the semantic representation of each word in the training set and determining whether the smallest distance was for the target representation.

## Results

### Word reading performance

After oral language training, the performance of the model was assessed. As expected, there was a considerable variation in model’s oral language performance due to the manipulation of amount of exposure and vocabulary size. For the meaning naming task (SP), the accuracy rate ranged from 44.73 to 100% (M = 90.01%, SD = 12.34%). For the oral vocabulary comprehension task (PS), the accuracy rate ranged from 38.90 to 99.75% (M = 89.50%, SD = 15.27%). With respect to reading, the variation in model’s performance was relatively small, ranging from 99.69 to 100% (M = 99.90%, SD = 0.06%) for reading aloud (OP) and from 91.52 to 99.08% (M = 96.65%, SD = 1.59%) for written word comprehension (OS). The results demonstrated that the model with different levels of oral vocabulary knowledge was able to learn to read and achieved reasonably high performance.

### Nonword reading performance

The model was then assessed to see if it could generalise to read previously unseen words. The model was tested on two sets of nonwords including 86 pseudowords taken from Glushko^3^ and 80 pseudowords taken from McCann and Besner^6^. With different levels of oral language skills, the nonword reading performance of the model varied between 74.19 and 85.22% (SD = 2.39%). The best performance produced by the model with high proficiency in oral language skills was comparable with that of Harm and Seidenberg^4^, whose model with high proficient oral language skills correctly pronounced 86.67% of the same set of nonwords. The present model was thus able to pronounce novel words effectively.

### Oral language and psycholinguistic reading effects

Next, the model was examined to see if it could reproduce a key range of psycholinguistic effects typically observed in human readers^39,40^ during word reading including frequency, orthographic neighbourhood, imageability, consistency, and the interaction between frequency and consistency. Additionally, the influence of oral language skills on word reading^8,14,26,28,41–43^ was also investigated. In the model, reading aloud was simulated by mappings from orthographic to phonological representations^4^. To compare the simulation results to the behavioural findings observed in mega studies^39,40^, a series of linear mixed-effect models (LMM) was conducted. LMMs were fit using the *lme4* package in R (version 4.1.1, 2021). The sum squared error (SSE) for each word that the model pronounced correctly was used as a dependent variable, where higher SSE produced by the model was conceived as slower response times (RTs) in behavioural studies^7,9^. The predictors were word frequency (WF), orthographic neighbourhood size (ONS)^2^, rime consistency (RC)^3,44^, imageability (IMG)^45^, oral vocabulary exposure (OVE), and oral vocabulary size (OVS). Words that the model misread, and outliers (> 2 standard deviations from the mean), were discarded. Outliers were removed because they could potentially dominate the outcome in LMM^46^. Additionally, following previous simulation work with regression analysis or LMM^23,28^, words without measures for all psycholinguistic variables were not considered. The data preprocessing procedures removed 0.043% of the observation points, leaving 621,671 observation points for analysis. To obtain standardised coefficients in LMM, all the variables including phonological SSE and the predictor variables were scaled^47^. That also resolved the issue of some predictor variables being on very different scales. As a baseline, an LMM model was constructed with item and model version as random factors, with WF, ONS, RC, IMG, and WF x RC as fixed factors, and with phonological SSE as the dependent variable. To investigate the unique effects of OVE and OVS, both variables were then included in the baseline model as fixed factors. That resulted in a significant improvement of model fit, χ^2^(2) = 1121.1, *p* < 0.001, compared to the baseline model. The result is shown in Table 1. The resulting patterns of WF, ONS, RC, IMG were in high accordance with previous mega studies^39,40^. Words were processed more quickly if they were higher in frequency, higher in imageability, higher in spelling-to-sound mappings, and higher in orthographic neighbourhood size. Moreover, the consistency effect was moderated by frequency. Critically, both OVE and OVS were significant predictors highlighted in bold in Table 1, in which larger oral exposures and vocabulary sizes were associated with quick responses (i.e., lower phonological SSE), consistent with previous studies^43^.

**Table 1:** Linear mixed-effect model fitted to phonological SSE produced by the model. All predictors were scaled.

	Standardised coefficient	*t*	95% Confidence Interval
WF	− 0.11	− 21.71	(− 0.12, − 0.10)
ONS	− 0.05	− 9.48	(− 0.06, − 0.04)
RC	− 0.09	− 16.67	(− 0.10, − 0.08)
IMG	− 0.05	− 10.25	(− 0.06, − 0.04)
WF*RC	0.05	10.06	(0.04, 0.06)
**OVE**	− **0.04**	− **30.77**	**(**− **0.04, **− **0.03)**
**OVS**	− **0.02**	− **13.23**	**(**− **0.02, **− **0.01)**

An effect was considered significant at the *p* < 0.05 level if its t-value was greater than 1.96^46^.

*WF* word frequency, *ONS* orthographic neighbourhood size, *RC* rime consistency, *IMG* imageability, *OVE* oral vocabulary exposure, *OVS* oral vocabulary size.

### Individual-level analysis

Next, the critical test of the present study was to investigate whether OVE and OVS could affect individual differences in the model’s reading in terms of the reliance on semantic access. The SR measures were computed using two approaches: (a) to evaluate the model in a way that was close to behavioural settings, following Woollams et al.^10^, the degree of SR during reading aloud exception words in the model was computed for each of 120 simulations of the model; (b) to directly investigate the model’s reliance on alternative reading pathways, a division of labour (DoL) technique^48,49^ was applied to reveal the use of the semantic pathway relative to that of the phonological pathway during reading. Both SR indices were evaluated by their predictabilities on reading aloud and nonword reading performance observed in Woollams et al.^10^. In line with the behavioural finding, it was expected that the model relying more on the OS pathway (i.e. high SR scores) would produce slower responses in both the reading aloud and nonword reading tasks. The observed effects would be modulated by spelling-to-sound consistency, especially for words. Lastly, the relationships between OVE, OVS, and the two SR measures were investigated.

#### Measuring SR based on the psycholinguistic approach

Woollams et al.^10^ quantified SR by measuring the effect of consistency (inconsistent-consistent) for words with low imageable concepts, termed *EoCSR*. To compute the EoCSR score in the model, the words in the training set were split into three quantiles based on the imageability measure^45^, labeling low (M = 2.70, SD = 0.55), medium (M = 4.20, SD = 0.39) and high (M = 5.91, SD = 0.56) imageability groups. Then, for each of the 120 simulations of the model, a regression analysis on the low imageable items was conducted with consistency as a predictor, and the model’s phonological SSE was used as a dependent variable. Log frequency and orthographic neighbourhood size were included as control variables.

**Figure 2:**
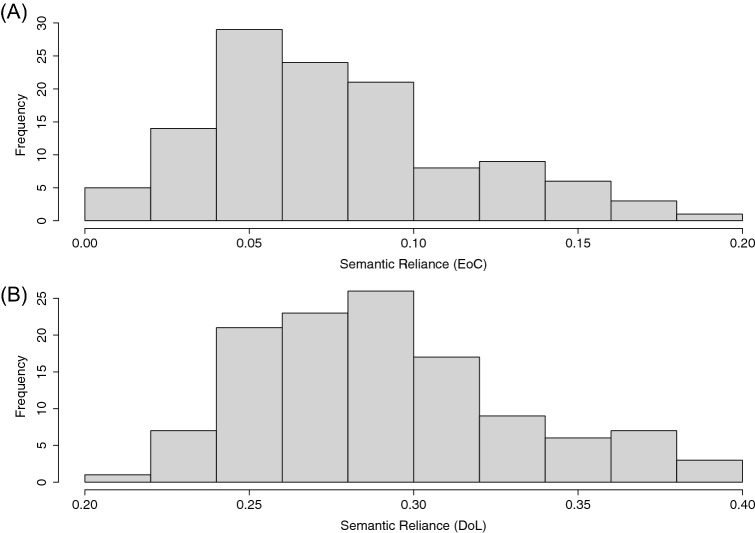
The distribution of semantic reliance based on the effect of consistency (EoC) approach (**A**). The distribution of semantic reliance based on the division of labour (DoL) approach (**B**).

Words that the model misread or without all psycholinguistic measures or outliers (> 2SD) were discarded. The resulting correlation coefficient of the consistency effect (consistent-inconsistent) was reversed and taken as SR, as in Woollams et al.^10^. As in Fig. 2A, the EoCSR scores among different simulations of the model varied between 0.1854 and 0.0034 (M = 0.075, SD = 0.039), demonstrating that the model with varying amounts of oral exposure and vocabulary sizes had different degrees of reliance on semantic information when reading words.

#### Measuring SR based on the division of labour approach

Note that the psycholinguistic effect approach to measuring SR (i.e. EoCSR) used the consistency effect when reading exception words as a lens into the utilisation of the OP and OS pathways in the human reading system. Though, in the model, the use of the OP and OS pathways can be more directly investigated by using a DoL technique^48,49^. Thus, in this section, SR in the model was computed by using the ratio of the activations contributed from OS versus OP reading pathways, term *DoLSR*. Specifically, the activation from orthography to phonology (OP) and the activation from orthography to semantics (OS) were computed. The activation was measured by first computing the absolute input to all the units in the target layer (phonology or semantics), and then the average score was taken. Although the input that a target unit received could be either positive or negative, the absolute value of the activations along the pathway was used, regardless of the sign to reflect the change in unit activation along each of the pathways in the model. The activation was taken at the last training time sample. Then, DoLSR was computed by dividing the OS activation by the sum of the OS and OP activations. The procedure was done for each of the 120 simulations with varying oral vocabulary sizes and amounts of exposure. As in Fig. 2B, the DoLSR ranged from 0.2179 and 0.397 (M = 0.291, SD = 0.0401). Taken together, the different degrees of SR associated with varying amounts of oral exposure and vocabulary sizes could be effectively captured by different approaches to measuring semantic reliance in the model. The correlation between EoCSR and DoLSR was significant at a medium level, 0.3592, *t*(118) = 4.18, *p* < 0.001, illustrated in Fig. S1.

### The prediction of SR on reading aloud and nonword reading

**Figure 3:**
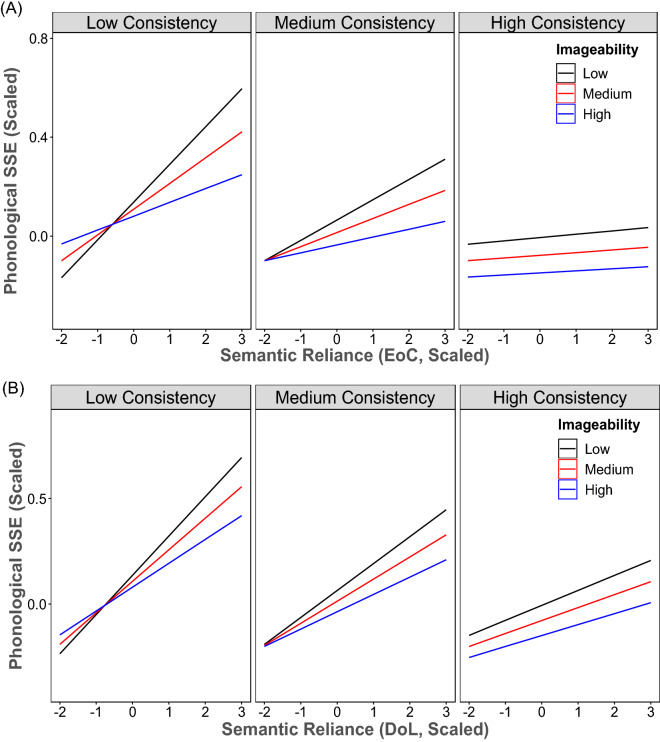
The three-way interaction between EoCSR (**A**) or DoLSR (**B**), imageability, and consistency in the reading aloud task. EoCSR: semantic reliance based on the effect of consistency; DoLSR: semantic reliance based on the division of labour.

Having computed the two SR indices, it was crucial to investigate whether they could indeed replicate the behavioural patterns of the reading aloud and nonword reading tasks observed in Woollams et al.^10^. For reading aloud, the key behavioural finding was the effect of SR being modulated by imageability and consistency, wherein readers with high SR showed a larger imageability effect than those with low SR, especially for inconsistent words. To test the model, four sets of stimuli were taken from Woollams et al.^10^ (Appendix A) including high-imageability consistent words, low-imageability consistent words, high-imageability inconsistent words, and low-imageability inconsistent words. Each stimulus set consisted of 40 words. Data cleaning procedures were identical to those in the previous section. The final data set consisted of 17,566 observations for further analysis. Two LMM models were constructed with item and model version as random factors, with imageability by consistency by EoCSR or DoLSR as fixed factors separately, and with phonological SSE as the dependent variable. All variables were scaled. For the LMM analysis of EoCSR, the results showed that EoCSR significantly predicted phonological SSE, *β* = 0.0573, *t* = 4.294, as well as consistency, *β* = − 0.0931, *t* = − 2.658. Imageability was not a significant predictor. Additionally, the two-way interactions between EoCSR and consistency, *β* = − 0.0467, *t* = − 6.789, and between EoCSR and imageability, *β* = − 0.0253, *t* = − 3.683 were significant while the interaction between consistency and imageability was not. Critically, the three-way interaction between EoCSR, imageability, and consistency was significant, *β* = 0.02287, *t* = 3.501. As illustrated in Fig. 3A, the imageability effect was stronger for the models with higher SR than those with lower SR, especially for reading inconsistent words.

For the LMM analysis of DoLSR, the results showed that DoLSR was a significant predictor, *β* = 0.1051, *t* = 9.973 so did consistency, *β* = − 0.0928, *t* = − 2.65. Imageability was not a significant predictor. The two-way interactions between DoLSR and consistency, *β* = − 0.0439, *t* = − 6.405, and between DoLSR and imageability, *β* = − 0.0229, *t* = − 3.335 were significant while the interaction between consistency and imageability was not. Moreover, the three-way interaction between DoLSR, imageability, and consistency was significant, *β* = 0.0134, *t* = 2.062. The resulting three-way interaction (Fig. 3B) was similar to that of EoCSR, assimilating the behavioural finding of readers with higher SR having a larger imageability effect than those with lower SR in particular for reading inconsistent words.

For nonword reading, the model was tested on the nonword set including 80 consistent nonwords and 80 inconsistent nonwords taken from Woollams et al.^10^ (Appendix C). Data cleaning procedures were identical to those applied previously, leaving 15,913 observations for nonword reading. Again, two LMM models were constructed with item and model version as random factors, with consistency by EoCSR or DoLSR as fixed factors separately, and with phonological SSE as the dependent variable. The LMM result of EoCSR showed both EoCSR, *β* = 0.9122, *t* = 2.797, and consistency (inconsistent-consistent), *β* = 0.4387, *t* = 5.391, significantly predicted the nonword reading performance. The interaction between EoCSR and consistency was not significant. Similar results were obtained in the LMM result of DoLSR. The result demonstrated that DoLSR, *β* = 0.6395, *t* = 1.987, and consistency (inconsistent-consistent), *β* = 0.3451, *t* = 2.613 contributed significantly whereas their interaction did not. Both results suggested that the models with higher SR produced more phonological SSE in nonword reading compared to those with lower SR, congruent with the behavioural data reported in Woollams et al.^10^.

### The relationships between OVE, OVS and SR

**Figure 4:**
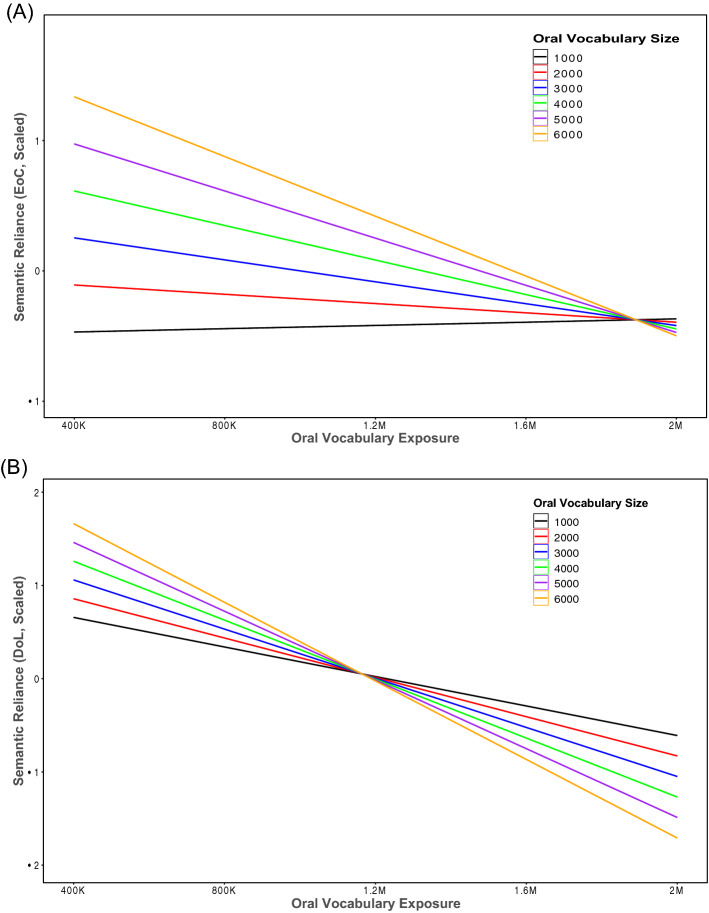
The two-way interaction between oral vocabulary exposure and oral vocabulary size on predicting EoCSR (**A**) or DoLSR (**B**). *EoCSR* semantic reliance based on the effect of consistency, *DoLSR* semantic reliance based on the division of labour.

The simulation results reported in the previous sections have demonstrated varying OVE and OVS could lead to different degrees of SR in the model of reading. Here the relationship between OVE, OVS, and the SR indices was directly investigated by using a series of simple regression analyses and structural regression analyses. For the simple regression analysis, both OVE, OVS, and their interaction term were employed as predictors, and SR was used as the dependent variable. The analysis was done separately for EoCSR and DoLSR. All predictors were scaled in order to make predictors and the dependent variable on a similar scale. The simple regression model of EoCSR produced an *R*^*2*^ value of 23.2% (Adjusted *R*^*2*^ = 21.2%), *p* < 0.001. Both OVE and OVS were significant predictors, *β* = -0.3076, *p* < 0.001, and *β* = 0.2872, *p* < 0.001, respectively. Critically, there was a significant interaction (Fig. 4A) between OVE and OVS in predicting the variance of EoCSR, *β* = -0.2356*, p* = 0.0047. As for DoLSR, the simple regression model produced an *R*^*2*^ value of 74.3% (Adjusted *R*^*2*^ = 73.6%), *p* < 0.001. OVE was a significant predictor, *β* = − 0.8231, *p* < 0.001 while OVS was not. Additionally, the interaction between OVE and OVS was significant, *β* = − 0.2564, *p* < 0.001 (Fig. 4B). The interaction patterns generated from EoCSR and DoLSR were similar. When OVE was limited, larger OVS led to larger SR scores. However, the SR scores decreased with the increase in oral vocabulary exposure. A reverse pattern of the interaction was seen more clearly in the result of DoLSR.

The regression results showed that both OVE and OVS could explain a significant portion of the variance of EoCSR and DoLSR. Though the magnitude was different (21.2% versus 73.6%). Further analysis was conducted to investigate to what degree the effects of the EoCSR and DoLSR on predicting the performance of the model in the reading aloud task might be driven by OVE and OVS. To address a potential causal relationship, two structural regression analyses were conducted using lavaan package in R^50^, with OVE, OVS and their interaction as measured variables, with either EoCSR or DoLSR as a latent variable and with model performance on reading aloud (i.e. the phonological SSE) as a dependent measured variable. Additionally, the possibility of OVE and OVS directly contributing to model performance on reading aloud was also examined.

**Figure 5:**
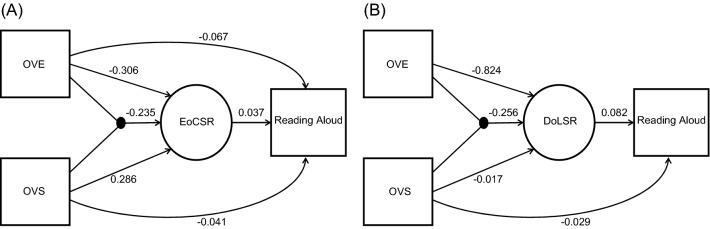
Structural regression analysis of model performance in the reading aloud task with OVE, OVS, and their interaction as measured variables and with (**A**) EoCSR or (**B**) DoLSR as a latent variable. *OVE* oral vocabulary exposure, *OVS* oral vocabulary size, *EoCSR* semantic reliance based on the effect of consistency, *DoLSR* semantic reliance based on the division of labour.

For EoCSR, a structural regression analysis was constructed with maximum likelihood as a method of estimation. The fit of the model was estimated using various goodness-of-fit measures including comparative fit index (CFI) = 1, root mean squared error of approximation (RMSEA) = 0 and tucker-lewis index (TLI) = 1. According to Hu and Bentler^51^, the CFI value of 0.95 or greater indicates a close fit. The RMSEA value of 0.06 or less indicates a close fit. The TLI value of 0.95 or greater indicates a close fit. Figure 5A illustrates the structural regression analysis of EoCSR on predicting model performance in the reading aloud task with standardised beta weights. All paths presented in the model were significant at *p* < 0.05. The covariance of the variables and residual variance were by default modelled in order to improve the fit of the model, but they were not depicted. The result demonstrated that both OVE (*β* = − 0.306, *p* < 0.001) and OVS (*β* = 0.286, *p* < 0.001) significantly predicted EoCSR. The interaction between OVE and OVS (*β* = − 0.235, *p* < 0.001) was also a significant predictor of EoCSR. The results were consistent with that of the simple regression analysis. Importantly, EoCSR significantly predicted phonological SSE produced by the model in the reading aloud task (*β* = 0.037, *p* < 0.001). Additionally, both OVE (*β* = − 0.067, *p* < 0.001) and OVS (*β* = − 0.041, *p* < 0.001) significantly predicted phonological SSE, but the interaction term (*p* > 0.05) did not. For DoLSR, another structural regression analysis was conducted. The result showed a close fit (CFI = 1, RMSEA = 0, and TLI = 1). Figure 5B illustrates the structural regression analysis of DoLSR on predicting model performance in the reading aloud task with standardised beta weights. The result demonstrated that OVE (*β* = − 0.824, *p* < 0.001), OVS (*β* = − 0.017, *p* < 0.001), and their interaction term (*β* = − 0.256, *p* < 0.001) all significantly predicted DoLSR, consistent with the simple regression analysis. DoLSR in turn was able to reliably predict phonological SSE produced by the model (*β* = 0.082, *p* < 0.001). Moreover, OVS (*β* = − 0.029, *p* < 0.001) was a significant predictor in accounting for phonological SSE, but neither OVE nor the interaction term did.

## Discussion

Individual differences in reading have been consistently reported for both adults and children^1,10,14–19^. The origins of individual differences, however, remain largely under investigation, possibly because factorising individuals’ capability and determining their causal relations with reading performance present a challenge. Theoretical proposals on the potential origins of individual differences in reading can be tested for their adequacy in the computational modelling of reading. Here, by extending the well-established triangle models of reading^4,28^, the critical issue of how oral vocabulary knowledge influences individual differences in reading in terms of the degree of SR was investigated.

The simulation results first demonstrated that the triangle model of reading with varying oral vocabulary skills was able to reproduce a range of standard reading effects including frequency, consistency, orthographic neighbourhood size, imageability, and the interaction between frequency and consistency. The result was in high accordance with behavioural reading data commonly reported in existing literature^39,40^. Additionally, the reading performance of the model was significantly predicted by oral vocabulary exposure and oral vocabulary sizes, demonstrating the influences of oral vocabulary knowledge on reading development^8,14,26,28,41–43^. By using both the behavioural-based and computational-based approaches to quantify SR (i.e., EoCSR and DoLSR) in the model, the result demonstrated that varying oral vocabulary skills could lead to different degrees of reliance on the alternative reading pathways in the reading system. Importantly, both EoCSR and DoLSR were able to predict the performance of the model in both reading aloud and nonword reading, as observed in Woollams et al.^10^. Further regression analyses revealed that both OVE and OVS directly contributed to EoCSR and DoLSR, and in turn, the two SR measures were able to predict the performance of the model on reading aloud, albeit to a different degree.

### The relationship between SR and reading performance

A previous behavioural study has demonstrated that readers with higher SR responded slower in the reading aloud task compared to those with lower SR^10^. The result was corroborated by a recent neuroimaging study, demonstrating that readers with high SR compared to those with low SR showed larger activations in the ATL associated with semantic processing and lower activations in the PCG associated with phonological processing^5^. The present simulations also produced a similar pattern, in which the model with high SR produced more phonological SSE in reading aloud relative to that with low SR. In the model, when the OP pathway was less used than the OS pathway for reading (i.e. high SR), more phonological SSE was observed. Furthermore, in line with Woollams et al.^10^, the simulation results also demonstrated that the effect of SR was modulated by word properties such as imageability and consistency. As illustrated in Fig. 3, the effect of SR was the strongest for low imageability words in the low consistency condition. Interestingly, for nonword reading, the effect of SR was found but it was not influenced by consistency. These simulation results suggest that skilled readers’ naming speeds are affected by a combined factor of individuals’ reliance on the use of alternative reading pathways and stimulus properties. However, the stimulus properties are more important for words than for nonwords, presumably because the readers are more sensitive to semantic and/or phonological properties elicited by the orthographic structure of word forms (i.e. words) compared to letter strings (nonwords).

The finding that SR predicts word and nonword reading performance seems straightforward because both tasks require converting orthography to phonology. Though in the model, phonology could also be accessed indirectly via the OS pathway and then the SP pathway. The process was not as efficient as direct access via the OP pathway, consistent with previous behavioural and computational investigations^24,52^. Moreover, according to the Simple View of Reading (SVR^53^), reading comprehension is a combination of reading fluency and oral language skills. Thus, reading fluency, the decoding of orthography to phonology, is a critical process for reading comprehension. Particularly in early literacy development, learning to read with instruction focusing on the OP mappings, but not on the OS mappings, has the benefit of transferring to word comprehension^52^. Thus, it might be expected that the use of the OP pathway would be useful not only for phonological access (e.g., reading aloud) but also for semantic access (e.g., written word comprehension). Siegelman et al.^14^ demonstrated that children with high sensitivity to OP regularities achieved higher performance on a range of reading tasks, including letter-word identification, word attack, and passage comprehension. Future work can be conducted to investigate whether a similar result can be obtained for written word comprehension in skilled readers and in the computational model of reading.

### Oral vocabulary knowledge as a source of individual differences in reading

A key objective of the present study was to investigate whether oral vocabulary knowledge could predict individual differences in the degree of SR in the model when other potential factors (reading experience and relative computational capacity) were controlled for. The simulation results demonstrated that oral vocabulary knowledge indeed contributed uniquely to the variance of EoCSR or DoLSR, consistent with the behavioural investigation by Siegelman et al.^14^. However, the present study went beyond previous research by showing that the influence of oral vocabulary size was modulated by oral vocabulary exposure. A large amount of exposure to a large vocabulary size would lead to a small SR, which means the OP pathway can be efficiently used. By contrast, limited exposure to a large vocabulary size would lead to an increased reliance on the OS pathway. This finding provides computational evidence that oral vocabulary knowledge could predict individual differences in reading. Critically, the influence of oral vocabulary knowledge was not additive but interactive, reflected in the amount of exposure to vocabulary size. Thus, in the model, both quantity and diversity of exposure are related to individual differences in reading.

Moreover, once the influence of oral vocabulary knowledge on SR was considered, SR was able to predict the reading outcome of the model. However, the predictability was larger for DoLSR than for EoCSR (0.082 versus 0.037 in Fig. 5). It is likely that the DoLSR was able to better characterise the relative use of the OP and OS pathways in the model while the measure of EoCSR was dependent on the inference of reading effects. More importantly, however, the investigations of the two SR measures resulted in largely similar resulting patterns with respect to their predictivities on reading performance and their relationship with oral vocabulary variables.

### Limitations and future work

Lastly, the present simulation results have demonstrated the influence of oral vocabulary knowledge on individual differences in reading, and the effect is above and beyond other commonly considered factors such as reading experience and relative computational capacity for reading processing. While it is important to investigate the isolated effect of oral vocabulary knowledge on individual differences in reading as shown in the present study, the processes of learning to read are dynamic rather than static. The development of oral language knowledge might be refined with the accumulation of reading experience^54^. The interactive process might also vary depending on individuals’ computational capacity in the reading system^9,21,22^. Thus, future work could be conducted to simultaneously explore the impact of reading experience, relative computational capacity, and oral vocabulary knowledge in the computational model of reading. Another significant point is that in the model oral vocabulary knowledge was operationalised in terms of oral vocabulary size and oral vocabulary exposure. The two measures especially oral vocabulary size are commonly used to provide a coarse measure of lexical knowledge because they are easily administrated^1^. However, high-quality representations of words are generally acquired in a gradual rather than a discrete way. As is evident, Andrews and collaborators^1,16^ demonstrated that orthographic precision could account for variability in individual differences in skilled word identification, and that is above and beyond vocabulary level of lexical quality. In the present study, the approach using the vocabulary level of lexical quality was adopted. With extensive exposure, the model would have developed an averaged high degree of fidelity for this experienced vocabulary. However, there might be some variability in the precision and coherence of the representations within the vocabulary, which might not be well characterised by simple measures of the amount of exposure and vocabulary size used in the present study. The present study is the first step in investigating the impact of oral vocabulary on individual differences in reading in the triangle model of reading. Future work can further explore how the precision and coherence of the representations affect individual differences in the degree of the SR.

## Conclusion

In conclusion, using a computational model of reading, the present study has established a direct link between oral vocabulary knowledge, particularly the amount of exposure to oral vocabulary size, and the degree of SR in the reading system. The observed effect of oral vocabulary knowledge on individual differences was unique and beyond reading experience and relative computational capacity for reading processing. Overall, the findings of the present study highlight that early differences in oral language experience can lead to subsequent variability in learning to read.

## Supplementary Information

Supplementary Figure S1.

## Data Availability

The modelling data and analysis scripts will be downloadable from the author’s GitHub: https://github.com/yaningchang/.
