# Supplementary material for: The influence of oral vocabulary knowledge on individual differences in a computational model of reading
Source: Sci Rep. 2023 Jan 30;13:1680. doi: 10.1038/s41598-023-28559-3 (PMC9886906; doi:10.1038/s41598-023-28559-3)
Supplement: Supplementary file 1 — Supplementary Figure S1. [file 41598_2023_28559_MOESM1_ESM.docx]

**Supplementary**

The influence of oral vocabulary knowledge on individual differences in a computational model of reading

Figure S1. The scatterplot of the correlation (*r* = 0.3592, *t*(118) = 4.18, *p* < 0.001) between semantic reliance based on the effect of consistency (EoC) and semantic reliance based on the division of labour (DoL).
